# A prospective clinical trial on the influence of a triamcinolone/demeclocycline and a calcium hydroxide based temporary cement on pain perception

**DOI:** 10.1186/1746-160X-8-9

**Published:** 2012-03-13

**Authors:** Brita Willershausen, Ines Willershausen, Vicky Ehlers, Adriano Azaripour, Benjamin Briseño

**Affiliations:** 1 Department of Operative Dentistry, University Medical Center of the Johannes Gutenberg University Mainz, Augustusplatz 2, 55131 Mainz, Germany

**Keywords:** Clinical study, Triamcinolone/demeclocycline, Calcium hydroxide based temporary cement, Post-operative pain perception

## Abstract

**Introduction:**

The aim of this clinical trial was to compare the degree of short term post-operative irritation after application of a triamcinolone/demeclocycyline based or a calcium hydroxide based provisional cement.

**Methods:**

A total of 109 patients (55 female and 54 male; mean age: 51 ± 14 years) with primary or secondary dentinal caries were randomly assigned to the two treatment groups of this biomedical clinical trial (phase III). Selection criteria were good systemic health and treated teeth, which were vital and showed no symptoms of pulpitis. Up to three teeth were prepared for indirect metallic restorations, and the provisional restorations were cemented with a triamcinolone/demeclocycyline (Ledermix) or a calcium hydroxide (Provicol) based material. The intensity of post-operative pain experienced was documented according to the VAS (4, 12, 20, 24, and 82 h) and compared to VAS baseline.

**Results:**

A total of 159 teeth were treated (Ledermix: 83 teeth, Provicol: 76 teeth). The minor irritation of the teeth, experienced prior to treatment, was similar in both groups; however, 4 h after treatment this value was significantly higher in the Provicol group than in the Ledermix group (p < 0.005, t-test). After 12 h, the difference was no longer significant. The number of patients taking analgesics for post-treatment pain was higher in the Provicol group (n = 11/53) than in the Ledermix group (n = 3/56).

**Conclusions:**

The patients had no long term post-operative pain experience in both groups. However, within the first hours after cementation the sensation of pain was considerably higher in the Provicol group than in the Ledermix group.

## Introduction

During cavity preparation pulpal inflammatory reactions should be avoided. Unpleasant sensory experiences and acute pain, which in many cases can go along with pulpal irritation up to an inflammation, may have various causes such as bacterial infections or chemical, physical or traumatic events. Cavity preparations, especially extensive prosthodontic preparations with a considerable removal of dentine, can be considered as particularly traumatic. Various authors have investigated the possible impact of prosthodontic preparations like full or partial crowns, involving an extensive loss of dentine, on the pulp tissues [1-3]. They reported that after an observation period of five years approximately 5% of the prepared teeth showed periapical radiological changes. Moreover, according to Bergenholtz et al. [4], necrosis occurs more frequently (15%-32%) in abutment teeth, especially when considering observation periods of more than 5 years. Cheung et al. [5] report similar results, and they observed that necrosis in these types of teeth occurs more frequently in maxillary anterior teeth.

Hodosh et al. [6] examined temporary interim cements for the cementation of provisional crowns regarding the aspect of a possible reduction of incidence and severity of pain after tooth preparation. It was shown in this study that a temporary cement, containing 4% potassium nitrate (KNO_3_) in a zinc oxide-eugenol (ZOE) base cement, was significantly more effective in reducing pain after tooth preparation than a cement containing only ZOE.

Glucocorticoids are drugs, frequently employed to avoid or reduce inflammation occurring after injuries or traumatic dental procedures. In addition, they can inhibit the progression of an inflammation, which would ultimately lead to necrosis of the pulp tissue. The anti-inflammatory effect of glucocorticoids, employed in the treatment of vital pulps after direct pulp capping, was first described by Rapoport et al. [7]. Later, glucocorticoids alone or in mixtures with antibiotics were applied as direct pulp capping agents or as liners in deep cavities to reduce pulpal pain [8-11]. Ledermix paste, developed by Schroeder [12], is a mixture of a glucocorticoid (triamcinolone) and an antibiotic (demeclocycline). Nowadays, two different types of Ledermix preparations are commercially available, which differ mainly in their consistency, and only slightly in their chemical composition. These preparations are Ledermix paste (1% triamcinolone, 3% demeclocycline-calcium) and Ledermix cement (0.7% triamcinolone, 3% demeclocycline-calcium).

According to various authors [13,14] the anti-bacterial and anti-inflammatory effects of Ledermix justify its employment, when inflammatory pulp conditions and apical lesions have been diagnosed. Schroeder [12] described the local application of glucocorticoids as a very effective therapy for pain reduction, especially in inflammatory pulp processes. Several authors [15-19] have discussed the potential anti-bacterial and anti-inflammatory effects of steroids, antibiotics, a combination of both agents, and calcium hydroxide in different investigations, often with controversial results. Hume et al. [15], using radio-labeled ^3^H-triamcinolone, observed that the constituents of Ledermix were released into dentinal areas close to the pulp. The authors claim that immunosuppression, a possible side effect of long term corticoid application, can favor the progression of pulp disease. In his review, Mohammadi [19] reported that the application of glucocorticoid steroids has been found to be effective in reducing pain following endodontic treatment. Sazak et al. [16] examined the teeth of dogs histopathologically for signs of pulp inflammation and formation of tertiary dentine after pulp exposure and application of calcium hydroxide/Ledermix or calcium hydroxide alone. After an observational period of 90 days, no difference was found between the two groups regarding the formation of reparative dentine. Ehrmann et al. [17] showed that the employment of Ledermix resulted in a reduction of endodontic post-treatment pain.

For the temporary cementation of provisional crowns, ZOE based cements, and those containing calcium hydroxide or eugenol, are most frequently used. A few studies can be found, dealing with the consistencies and retentive properties of various provisional cements [20-22] however, the aspect of pain reduction after tooth preparation was not examined. In these studies, either the retention of provisional crowns luted with different temporary cements [21] or the effect of the temporary cements on the bond strength of the final cementation with resin based luting cements to dentine [20,22] were examined.

The aim of this clinical trial was to observe and compare the degree of short term post-traumatic pain, caused by irritation during cavity preparation, after application of a triamcinolone/demeclocycline or a calcium hydroxide based temporary cement. Only vital teeth with primary or secondary carious lesions with an extensive loss of dentine, in need of indirect restorations and without any sign of pulpal inflammation were included. The provisional restorations were luted with Ledermix cement or Provicol. By means of specific pain diaries the patients recorded the intensity of post-operative pain experienced at different time points after the dental treatment, and in addition provided information about amount and frequency of intake of analgesic drugs.

## Materials and methods

### Ethical approval and patient consent

This clinical trial was approved by the Institutional Review Board of the University of Mainz, the Ethics Committee (Nr: 837.033.07 5568) and the Federal Institute for Drugs and Medical Devices (BfArM; Nr: 4032703). The study was conducted according to the principles of the Declaration of Helsinki in the revised form of Tokyo und Venice. Furthermore, the rules of the German Drug Law (AMG) were followed. All patients who met the selection criteria and were included in this examination were informed verbally and in written form about the risks and benefits of the study and consented to participate. The text of the written informed consent of the patients to participate in the clinical trial was attached to the study protocol. It was signed by the patients prior to enrolment in the study. One copy was given to the patient; the other stayed with the clinical investigator because of the need for confidentiality, and to have it as certificate for the patient's informed consent on hand at all times.

### Design of the study and inclusion/exclusion criteria

The study was conducted according to a 1-period, 2-parallel-groups, block-randomized design, and comparisons were made between the two patient groups.

The trial was carried out as an observer-blind, reference-controlled, mono-centric phase III study in patients after dental treatment of teeth with dentinal caries and/or defective filling margins, leading to a normal or considerable loss of hard tissue (dentine).

Inclusion criteria for this clinical trial were adult patients aged up to 75 years and good systemic health. The cardiovascular parameters as well as the patients' height and weight for the determination of the BMI index were determined and recorded to avoid possible general health complications. Exclusion criteria were pregnancy, constant medication within the last six months, mental illness, physical disabilities, neuropathies or severe cardiovascular disease, hypersensitivities to corticoids or tetracycline. All patients had one to three dentine carious lesions of primary or secondary origin, and an indirect metallic restoration (inlay/onlay, partial or full crowns) with a considerable loss of dentine was indicated. Prior to tooth preparation, all teeth had to be vital and show a negative reaction to percussion. Teeth were excluded, when the presence of a reversible or irreversible pulp inflammation was suspected.

Patients reporting a pre-treatment pain score of 50 or more on a VAS (Visual Analogue Scale) were excluded from the study. To fulfill the quality criteria of decision, n = 45 patients per group were required [23]. Assuming a drop-out rate of about 10%, 50 patients per group had to be included into the study.

### Coding and randomization of the patient collective

The randomization code was produced by SAM GmbH (Greifswald, Germany). Each patient was randomly assigned to the investigational or the reference drug, using a computer-generated random permutation procedure (SAS^® ^Proc PLAN). Randomization was performed in blocks. The size of the blocks was defined only in the randomization list. This list was not available to the investigator for the second visit (observer blind). One copy of the master randomization code was kept at SAM GmbH.

### Clinical procedures

For this clinical trial originally 235 patients of both genders were considered; however, only 110 patients met the inclusion criteria, and one patient dropped out due to an illness. The randomization of the patients was carried out after patient recruitment and enrolment in the study.

A total of 109 patients were enrolled in the study (55 female and 54 male). The patients were between 22 and 74 years of age (mean value: 51 ± 14 years). After randomization, the two patient groups were matched for age range, gender and type of restoration, as is shown in Table 1. Based on the process of randomization, in 56 patients (31 female, 25 male; aged 23-74 years) 83 teeth were treated with Ledermix cement, and in 53 patients (29 male and 24 female; aged 22-72 years) 76 teeth were treated with Provicol. Prior to the tooth preparation, the patients had to assess the pain perception of each respective tooth with the help of a visual analogous pain scale (VAS: 0-100). Five calibrated dentists with at least five years of experience carried out the different tooth preparations for crowns and other indirect restorations involving loss of dentine structure. The distribution of the types of final restoration after randomization is very similar in both groups (Table 1). A maximum of three teeth per patient were included in the study, the mean number of teeth treated per patient was 1.48 for the Ledermix group, and 1.44 for the Provicol group. In most cases, an extensive loss of dentine was observed and a self-adhesive core building material was used (Luxacore DMG, Hamburg, Germany). After the tooth preparations and cementation of the provisional restoration, the patients received a pain diary, which was explained in detail to all participants at the beginning of this clinical trial, and they had to complete it up to three days following the dental treatment. In addition to keeping the pain diary, all patients were questioned via telephone (on the first and third day after treatment) about their actual well-being and possible pain sensations. The patients were appointed for the definitive cementation of the indirect restoration 12 ± 3 days after the initial treatment (second office visit). At that appointment they were requested to bring along the pain diary. The results of the sensitivity and percussion tests and the documentation of eventual pain and intake of analgesics (Ibuprofen 400 mg, three times a day or less) were recorded. These data were transferred into an eCRF software (electronic Case Report Form/Greifswald, Germany). Subsequently, the temporary restorations were removed and the entire cavity was thoroughly disinfected with 0.1% chlorhexidine (Chlorhexamed Fluid, GlaxoSmithKline/München, Germany). Then the permanent restoration was luted with a self-adhesive universal luting cement (RelyX Unicem, 3 M ESPE, Seefeld, Germany). Twenty-eight ± 3 days after the first treatment, the patients were appointed for a control visit (third office visit). The tooth sensitivity, sensitivity to percussion, occlusion, as well as the patients' general conditions were recorded.

**Table 1:** Patients' data and type of restorations in the Ledermix and the Provicol groups

Number of patients n = 109	Ledermix group n = 56	Provicol group n = 53
Age (years) (mean ± SD; range)	53.3 ± 13.7; 23 -74	49.2 ± 14.6; 22-72
Males	25 (44.6%)	29 (54.7%)
Females	31 (55.4%)	24 (45.3%)
Number of teeth (n = 159)	83 (1.48 per patient)	76 (1.44 per patient)
Type of tooth preparation	42 (50.6%)	35 (46.1%)
Crowns	27 (32.5%)	21 (27.6%)
Partial crowns	14 (16.9%)	20 (26.3%)
inlay/onlay core restoration	54 (65.1%)	45 (59%)
Baseline pain (VAS: 0-100) (mean ± SD)	5.6 ± 8.8	4.3 ± 6.8

### Test materials

After the completion of the cavity preparation for an indirect restoration, the teeth received a temporary restoration made from a resin-based material (Protemp 4, 3 M ESPE, Seefeld, Germany). This temporary restoration was cemented with Ledermix (Riemser Arzneimittel AG/Greifswald, Germany) or with Provicol/C/QM (Voco GmbH/Cuxhaven, Germany). Ledermix cement contains demeclocycline and triamcinolone acetonide, while Provicol is a calcium hydroxide-based, eugenol-free temporary cement. The temporary restorations remained in place for up to 12 days (± 3), after which they were removed, and the teeth received their final restorations.

### Statistical analysis

The primary outcome variable is the difference (Δ) between the VAS scores after treatment compared to pre-treatment VAS scores. The arithmetic means, standard deviations and the corresponding two-sided 90% confidence intervals of these Δ-values were calculated. The assessment was done using the SAS^® ^package. The one sided-t-test was used for statistical evaluation (α = 0.05). All other variables were evaluated in the sense of an explorative analysis. All statistical tests that were carried out at secondary variables are strictly interpreted in an exploratory sense to generate hypotheses, not to decide on hypotheses.

## Results

A total of 109 patients were enrolled in the study (55 female and 54 male). The patients were between 22 and 74 years of age (mean value: 51 ± 14 years). Based on the process of randomization, in 56 patients (31 female, 25 male; aged 23-74 years) 83 teeth were treated with Ledermix cement, and in 53 patients (29 male and 24 female; aged 22-72 years) 76 teeth were treated with Provicol.

**Figure 1:**
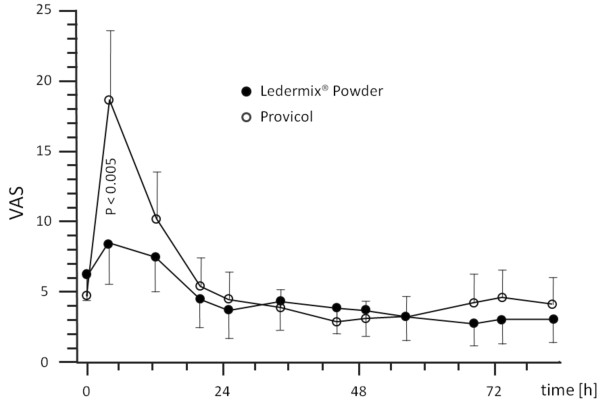
**VAS (score: 0-100) pain scale taken from the patients' diaries during the course of the first study days (mean ± CI, confidence interval)**.

There were no significant differences between the two groups with regard to age, gender and blood pressure values. Based on the performed randomization, one to three teeth were treated per patient. The tooth related evaluation of pain perception showed in the Provicol group at the beginning of the observation time (up to 12 hours) higher pain perception scores than in the Ledermix group. The greatest differences regarding the pain perception were observed 4 h after tooth preparation, when the patients in the Ledermix group showed statistically significant lower pain perception scores than those in the Provicol group (*p *< 0.005). After twelve hours, the pain perception scores in the Ledermix group were still lower; however, the difference was no longer statistically significant (Figure 1).

For the remaining observational period of three days, almost no differences were reported. The analysis of the pain diaries showed that patients in the Provicol group had a higher demand for analgesics. Only 3/56 patients from the Ledermix group took analgesics (each 1× Ibuprofen 400 mg). In contrast, 11/53 patients from the Provicol group took analgesics, four of them took more than 1 × Ibuprofen 400 mg during the first day, and four patients took analgesics for more than one day. During the entire observational period of 28 days, no severe pain was observed, all teeth remained sensitive and none showed a positive response to percussion indicating an irreversible pulpitis, and no tooth discoloration, based on the short term placement of Ledermix cement, was recorded. None of the teeth showed periradicular changes on the x-rays, and no endodontic treatment was performed. In addition, no long term post-operative pain experience was observed in both groups.

## Discussion

After tooth preparations, which involve a considerable loss of dentine, a rapid and efficient pain relief is of major importance. In the present prospective, randomized, observer-blind, reference-controlled phase III clinical study, the influence of Ledermix cement in reducing post-operative pain was compared to calcium hydroxide-based Provicol cement. Since its introduction by Schroeder [12], the application of Ledermix has been recommended mainly for endodontic treatment, and for the relief of pain induced by pulp irritation. The efficacy of its active agents has been controversially discussed. Different authors report the advantages of using Ledermix for pain reduction in endodontic procedures [10,17,19,24]. However, other authors were not able to report significant differences between Ledermix and calcium hydroxide after intraradicular application with regard to pain reduction [25,26]. To the best of our knowledge, there are only few studies reporting the efficacy of Ledermix as temporary cement on vital teeth, especially in cases of extensive crown and cavity preparations. Abbott et al. [27] report a beneficial effect of Ledermix in the treatment of cracked teeth with clinical signs of reversible pulpitis. In the present investigation, a possible reduction of post-traumatic pain by two temporary cements (Ledermix and Provicol) was investigated. An equally important aspect was the possible reduction of microorganisms, remaining behind after the removal of carious lesions. As pain is a subjective sensation and can easily be influenced, all patients were asked to keep a pain diary for a defined period of time after the treatment. Pierce et al. [28] were able to show histologically that the application of Ledermix resulted in a reduction of inflammatory external root resorption and thus exerted an inhibitory influence on rapidly progressing root resorptions in injured teeth. Glucocorticoids suppress cell-mediated immunity by directly inhibiting all steps within the eicosanoids synthesis. As a consequence, the steroidal component of the Ledermix is able to suppress the cell-mediated immunity [28]. Some authors raise concern that steroids might have an inhibiting effect on the hypophysical system. However, it could be shown that the maximal dose of glucocorticoids applied within the root canal has only a local effect and is therefore not high enough to exert a systemic effect [29].

The present study revealed that teeth, which received Ledermix cement, showed a significantly lower pain perception 4 h after tooth preparation, when compared to Provicol cement; however, after 24 h no significant difference between the two groups could be observed. Over an observational period of four weeks, no cases of tooth sensitivity loss were reported, and in addition, the patients had no long term post-operative pain experience in both groups. Both tested materials can be recommended as temporary cements.

## Competing interests

The authors declare that they have no competing interests.

## Authors' contributions

VE, IW and AA carried out the study and examined all patients. VE and IW performed the statistical analysis. BB participated in the design of the study. BW conceived of the study, and participated in its design and coordination. All authors read and approved the final manuscript.
